# Isolated traumatic retroclival hematoma: case report and review of literature

**DOI:** 10.1007/s00381-016-3098-y

**Published:** 2016-04-27

**Authors:** Ha Son Nguyen, Saman Shabani, Sean Lew

**Affiliations:** Department of Neurosurgery, Medical College of Wisconsin and Children’s Hospital of Wisconsin, 9200 West Wisconsin Ave, Milwaukee, WI 53226 USA

**Keywords:** Retroclival hematoma, Abducens nerve palsy

## Abstract

**Background:**

Retroclival hematomas are a rare entity. The pathology can be categorized into epidural hematoma or subdural hematoma based on the anatomy of the tectorial membrane. Frequently, the etiology is related to accidental trauma, though other mechanisms have been observed, including coagulopathy, non-accidental trauma, and pituitary apoplexy. There have been only 2 prior cases where both epidural and subdural hematoma co-present.

**Case presentation:**

An 8-year-old male was involved in a high-speed motor vehicle accident. He presented with a Glasgow Coma Score (GCS) of 14 with bilateral abducens nerve palsies. Computed tomography (CT) revealed a hemorrhage along the dorsum sella, clivus, and dens. Magnetic resonance imaging (MRI) demonstrated the retroclival hematoma in both the subdural and epidural space. At discharge, 19 days after the accident, the abducens nerve palsies had resolved without medical or operative intervention.

**Conclusion:**

Retroclival hematoma may present after trauma. Although most cases exhibit a benign clinical course with conservative management, significant and profound morbidity and mortality have been reported. Prompt diagnosis with close observation is prudent. Surgical management is indicated in the presence of hydrocephalus, symptomatic brainstem compression, and occipito-cervical instability.

## Introduction

Retroclival hematomas are rare and only represent a small subset of posterior fossa extra-axial hematomas, which as a whole constitute approximately 0.3 % of acute extra-axial hematomas [1, 2]. The pathology can be categorized into epidural hematoma (rcEDH) or subdural hematoma (rcSDH) based on the anatomy of the tectorial membrane. Most cases in the literature involve the pediatric population, though few cases have been reported in the adult population as well. Frequently, the etiology is related to accidental trauma, though other mechanisms have been observed, including coagulopathy, non-accidental trauma, pituitary apoplexy, and ruptured aneurysm. Still, some remain spontaneous without an identifiable cause [3–8]. We report a pediatric patient who sustained a retroclival hematoma (with both subdural and epidural components) after a motor vehicle crash and provide a review of the available English literature, emphasizing the pathophysiology of injury and the appropriate clinical management. There have been only 2 prior cases where both epidural and subdural hematoma co-present [9].

## Case presentation

An 8-year-old male was involved in a motor vehicle crash. He was sitting on the back seat along the driver side; his seat belt status was unknown. The vehicle was “T-boned” by another vehicle traveling 60 miles per hour. At the scene, patient exhibited a GCS 14. On presentation, his eyes were crossed, but he did not complain of diplopia until the following day. Because he was lethargic and confused, he was admitted to the ICU for close monitoring. He denied significant headaches, blurred vision, eye pain, or light sensitivity. Physical examination was significant for bilateral 6th nerve palsies.

**Fig. 1:**
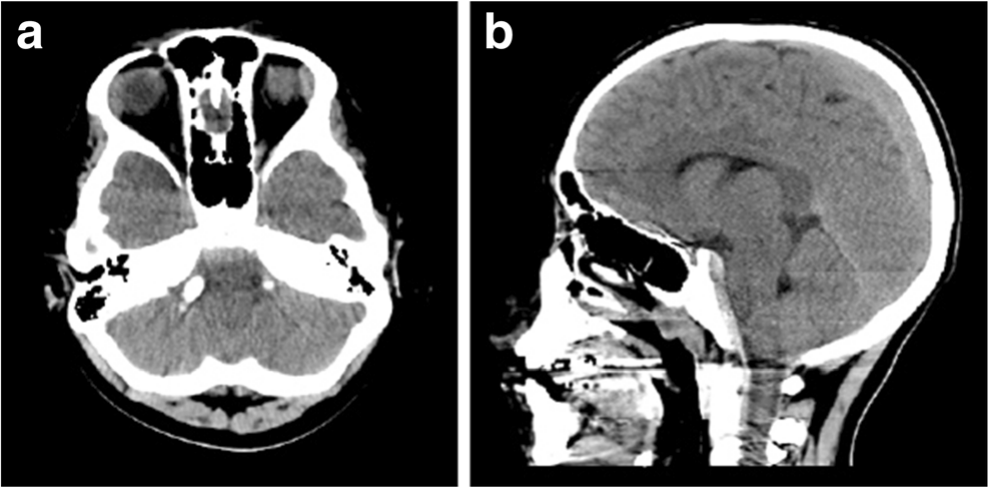
**a** Axial CT head demonstrates retroclival hematoma. **b** Mid-sagittal CT head demonstrates retroclival hematoma

**Fig. 2:**
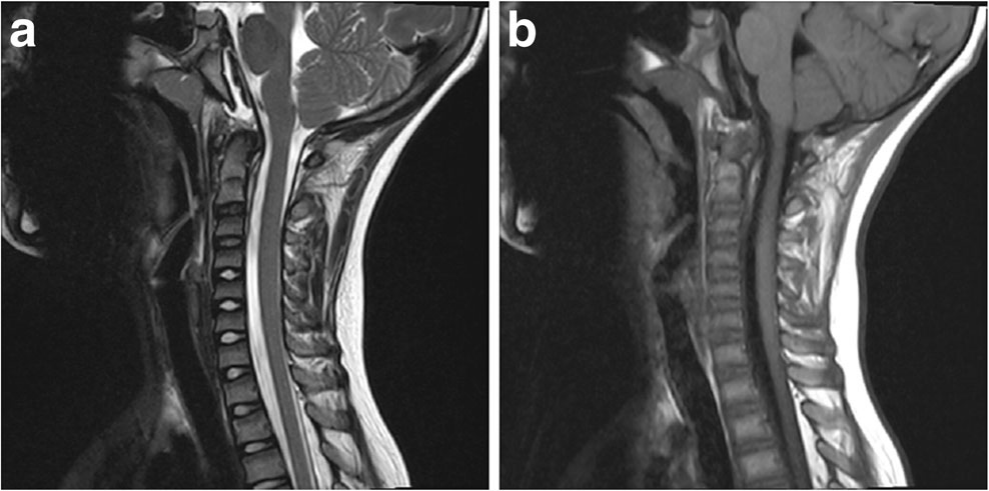
**a**, **b** Sagittal T2 and T1 MR demonstrate rupture of tectorial membrane, with hematoma both ventral and dorsal to the membrane. Epidural hematoma tracks to mid-body of the dens, while subdural hematoma tracks to inferior C3 body

CT of the head revealed a hemorrhage along the dorsum sella, clivus, and dens (Fig. 1a, 1b). MRI brain and cervical spine were obtained to evaluate the hematoma and the craniocervical junction for signs of instability; the retroclival hematoma appeared in the subdural space and epidural space; there was T2 hyperintensity in atlanto-occipital joints and blood along the tectorial membrane (Fig. 2a, 2b). Subsequently, cervical spine flexion/extension x-rays were obtained, which demonstrated no instability and the cervical collar was discontinued. The patient had a prolonged hospitalization due to a duodenal hematoma and associated feeding issues. At discharge, 19 days after the accident, he exhibited intact eye movements.

## Discussion

Retroclival subdural hematoma (rcSDH) has been reported less often than epidural hematoma (rcEDH). However, both can co-present, particularly in violent injuries [10]. Tables 1, 2, 3, and 4 summarize the available English literature. In the pediatric population, there have been 30 cases of rcEDH and 16 cases of rcSDH; in the adult population, there have been 8 cases of rcEDH and 21 cases of rcSDH. The tectorial membrane helps define the distinction between the epidural space and the subdural space, where the former is ventral to the membrane and the latter is dorsal to the membrane [11]. The tectorial membrane is the rostral continuation of the posterior longitudinal ligament, attached inferiorly to the posterior body of the axis and superiorly to the occipital bone along the clivus [11]. RcEDH are restricted by the boundaries of the membrane (that is, from the mid-portion of the clivus to the middle of the body of the axis); rcSDH are not restricted and can disseminate from the intracranial to the spinal subdural space [11]. The MRI (Fig. 2a, 2b) from our patient demonstrated stripping of the tectorial membrane, with focal areas of disruption; the ventral fluid collection tracking down to the mid body of the odontoid is consistent with an epidural hematoma; however, there is also a collection that exists posterior to the tectorial membrane and tracks more inferiorly to the posterior of the C3 body; this collection is consistent with a subdural collection.

**Table 1:** Literature review of pediatric rcEDH

Literature	Year	Age	Gender	Mechanism	Exam	Surgery?	Long-term deficits	Other features
Orrison^17^	1986	8 years	M	MVA while riding bike	GCS 3, polytrauma, blown pupils and no brain stem reflexes	Evacuation of parietal hematoma (not for RCH)	Died	Odontoid fracture, rupture of transverse ligament, brain stem contusion, pontine hemorrhage, 4th ventricle hemorrhage
Kurosu^22^	1990	11 years	F	MVA while crossing street	GCS 7, quadriparesis	No	Slight right arm paresis	Spheno-occipital synchondrosis’ diastasis
Papadopoulos^19^	1991	10 years	M	MVA while crossing street on bicycle	GCS 4, bilateral 6th, quadraparesis, shallow respirations	Evacuation of hematoma via posterolateral approach, then posterior fusion	None	AOD
Marks^38^	1997	8 years	F	MVA	GCS 6, quadriplegia, apneic	transoral evacuation, posterior stabilization	Mild left hemiparesis, able to walk unaided	AAD
Mizushima^34^	1998	8 years	M	MVA while crossing street	GCS 7, bilateral 6th, mild bilateral arm paresis	No	None	AAD
Suliman^21^	2001	16 years	M	MVA versus a tree	GCS 8, paresis of 9, 12 th cranial nerves, right hemiparesis	No	None	Left occipital condyle fracture
Yang^36^	2003	5 years	M	MVA while crossing street	GCS 7, poor spontaneous respiration, right side hemipareis/poor fine motor control	No	None	***
Agrawal^33^	2006	8 years	F	MVA	GCS 7, bilateral 6th, left 12th palsy	No	None	***
Paterakis^16^	2005	10 years	M	MVA	GCS 13, right 6th, right 9th cranial nerve, partial 7th	No	Minimal 6th palsy	Clival fracture
Guillaume^13^	2006	5 years	F	MVA versus tractor trailer	GCS 8, right gaze preference, right hemiparesis	No	Mild spastic quadriparesis	***
Guillaume^13^	2006	8 years	M	MVA	Confused but alert, following commands	No	None	***
Vera^20^	2007	5 years	F	MVA	GCS 3, fixed/dilated pupils/cardiorespiratory arrest/polytrauma/obstructive hydrocephalus	EVD	Died	AOD
Kwon^14^	2008	11 years	F	MVA	GCS 15, bilateral 6th palsy, uvula deviation to left, weak tongue	No	None	***
Tubbs^39^	2010	Mean 12 years	5 male and 3 female patients	MVA-related	Mean GCS 8	2 patients with stabilization	2 died, 4 patients are neurologically intact, 1 patient had a complete upper cervical spinal cord injury, 1 patient had mild bilateral abducens nerve palsy	2 AOD
Becco de Souza^32^	2011	8 years	F	MVA	GCS 15, bilateral 6th	No	None	***
McDougall^30^	2011	10 years	F	MVA	GCS 14, right 6th palsy	No	Minimal 6th nerve palsy	***
Tahir^12^	2011	12 years	F	MVA	GCS 11, right hemiparesis	No	Improving right hemiparesis	***
Silvera^9^	2014	2 months	F	Abuse	***	***	***	***
Silvera^9^	2014	1 months	M	Abuse	***	***	***	***
Silvera^9^	2014	13 months	M	Abuse	***	***	***	***
Silvera^9^	2014	30 months	F	Abuse (both SDH and EDH)	***	***	***	***
Silvera^9^	2014	1 months	F	Abuse (both SDH and EDH)	***	***	***	***
Dal Bo^3^	2015	2 years	M	Spontaneous, neck pain	NF	No	None	***

*GCS* Glasgow Coma Scale, *MVA* motor vehicle accident, *AOD* atlanto-occipital dislocation, *AAD* atlanto-axial dislocation, *** no data, *EVD* external ventricular drain, *RCH* retroclival hematoma, *SDH* subdural hematoma, *EDH* epidural hematoma, *M* male, *F* females; *NF* non-focal

**Table 2:** Literature Review of Adult rcEDH

Literature	Year	Age (years)	Gender	Mechanism	Exam	Surgery?	Long-term deficits	Other features
Tomaras^8^	1995	36	M	Spontaneous	GCS 15, left 7th nerve palsy	No	None	
Goodman^24^	1997	62	M	Pituitary apoplexy	Chiasmal syndrome	Pituitary resection	Improvement of chiasmal syndrome	Resection of hemorrhagic pituitary adenoma
Calli^27^	1998	42	M	Status post posterior fossa decompressive surgery for cerebellar infarct	***	Posterior fossa decompressive surgery, not for RCH	***	***
Khan^15^	2000	19	M	MVA	GCS 12, right 3rd palsy, dilated nonreactive right pupil failing, bilateral 6th palsy, right 7th palsy, bilateral conductive hearing deficit	No	Partial improvement right 6th and 3rd, recovery of left 6th. stable 7th paresis, no hearing deficits	Fracture of the posterior clinoid and clivus extending into the sphenoid sinus
Ratilal^31^	2006	26	F	MVA	GCS 13, bilateral 6th, bilateral V3 numbness, left 12th palsy	No	Mild diplopia on extreme lateral eye movements and left tongue deviation	***
Cho^7^	2009	36	M	Spontaneous (dilated cervical epidural veins)	NF	No	None	Bilateral supratentorial SDH, epidural venous engorgement
Datar^37^	2013	75	M	Tripped on rug, head trauma	NF	Posterior fusion	Died	oumadin coagulopathy
Perez^18^	2013	68	M	MVA	GCS 15	No	Died	Odontoid fracture, cardiorespiratory arrest

*GCS* Glasgow Coma Scale, *MVA* motor vehicle accident, ***no data, *RCH* retroclival hematoma, *SDH* subdural hematoma, *M* male, *F* females, *NF* non-focal

**Table 3:** Literature review of adult rcSDH

Literature	Year	Age (years)	Gender	Mechanism	Exam	Surgery?	Long-term deficits	Other features
Narvid^4^	2015	58	M	Spontaneous	NF	None	None	IVH
		64	F	Spontaneous	NF	None	None	***
		64	M	Spontaneous	Diplopia	None	None	IVH
		67	M	Spontaneous	Unresponsive in the Emergency Department	None	None	IVH
Azizyan^23^	2015	Mean 55	8 M, 2 F	Pituitary apoplexy	9 of 10 exhibited ophthalmoplegia	8 of 10 surgery for pituitary, did not address RCH	***	***
Mohamed^1^	2013	37	M	Pituitary apoplexy	Left 3rd, left temporal field cut, decreased visual acuity bilaterally	surgery for pituitary	Partial improvement in the patient’s third nerve palsy and visual acuity	***
Krishnan^28^	2013	59	F	Thrombocytopenia	Flexing both upper limbs to pain, Both plantars were extensor	None	Died	Left convexity SDH
Schievink^5^	2001	49	F	Spontaneous	NF	None	None	***
Sridhar^35^	2010	19	M	Fall from moving bus	NF	None	None	***
van Rijn^6^	2003	72	M	Spontaneous	Bilateral 6th, bilateral leg paresis	***	***	***
Kim^25^	2012	83	F	Pcomm aneurysmal rupture	*Confusion*	Coil embo for aneurysm	None	***
Brock^26^	2010	42	F	Infraclinoid aneurysm	3rd, 4th right paresis	Aneursym clipping	None	***

*GCS* Glasgow Coma Scale, *** no data, *IVH* intraventricular hemorrhage, *RCH* retroclival hematoma, *SDH* subdural hematoma, *M* male, *F* females, *NF* non-focal

**Table 4:** Literature review of pediatric rcSDH

Literature	Year	Age	Gender	Mechanism	Exam	Surgery?	Long-term deficits
Ahn^40^	2005	4 years	M	Fall, four-story window	Left side hemiparesis	None	None
Myers^29^	1995	17 years	M	Hemophilia, slipped on ice and hit head	Comatose, fixed dilated pupils, no brain stem reflexes		Died
Casey^2^	2009	18 years	M	Trivial head injury	GCS 13	None	None
Sridhar^35^	2010	18 years	M	Fall from two-wheeler	Bilateral 6th	Yes, evacuation of RCH	None
Silvera^9^	2014	3 months	M	Abusive	***	***	***
		1 months	F	Abusive	***	***	***
		3 months	M	Abusive	***	***	***
		1 months	M	Abusive	***	***	***
		36 months	M	Abusive	***	***	***
		30 months	M	Abusive	***	***	***
		7 months	F	Abusive	***	***	***
		7 months	F	Abusive	***	***	***
		3 months	M	Abusive	***	***	***
		4 months	F	Abusive	***	***	***
		4 months	M	Abusive	***	***	***
		30 months	F	Abusive	***	***	***

*GCS* Glasgow Coma Scale, *** no data, *RCH* retroclival hematoma, *M* male, *F* females, *NF* non-focal

The most common etiology is a traumatic event that induces hypermobility of the neck. Either hyperflexion or hyperextension can lead to soft tissue injury or fractures, causing a retroclival hematoma. The preponderance of reported pediatric cases relative to adult cases may be attributed to the anatomical differences at the craniocervical junction. Compared to adults, children possess certain features (large head-to-body proportion, small occipital condyles, shallow facet joints, and weak cervical muscles) that increase the mobility of the spine and augment the risk for injury [12, 13]. Disruption of the tectorial membrane (i.e., from its insertion into the clivus) can cause venous bleeding from the surrounding basilar venous plexus and dorsal meningeal branch of the meningohypophyseal trunk, leading to an epidural collection [11]. In children, the dura can be more easily detached from the bone, which makes them more vulnerable to forceful traction [14]. Clival fractures have been associated with rcEDH, likely due to bone bleeding as well as injury to the tectorial membrane [15, 16]. Similarly, odontoid fractures have been reported; dislocation of the dens can cause damage to the transverse ligament and traction to the tectorial membrane, prompting hemorrhage [17, 18]. Shearing forces may lead to rcSDH via rupture of the bridging petrosal and small veins near the foramen magnum; the tectorial membrane is usually unharmed, remaining attached to the clivus; this feature is an important characteristic which differs from rcEDH [11]. Other traumatic injuries associated with retroclival hematoma include atlanto-occipital dislocation [19, 20], atlanto-axial dislocation, rupture of the transverse ligament [17], fractures of the occipital condyles [21], spheno-occipital synchondrosis diastasis [22], brain stem contusion [17], and intraventricular hemorrhage [17].

There are a variety of non-traumatic causes of retroclival hematoma. A common etiology is pituitary apoplexy. Hemorrhage can spread through the diaphragm sella into the subdural space, constrained by the posterior arachnoid membrane of the prepontine cistern [1, 23]; on the other hand, a defect in the dorsum can permit blood flow into the epidural space [24]. Rare cases of rcSDH have been associated with aneurysmal rupture [25, 26]. Moreover, pressure changes (spontaneous intracranial hypotension [7] and posterior fossa decompressive craniectomy [27]), thrombocytopenia [28], and hemophilia [29] have been linked with rcSDH. Several cases have occurred spontaneously with negative work-up and no history of trauma [3–8].

Clinical presentation can be variable. Neurological impairment may be related to stretching, direct compression, or contusion of surrounding nerves and brain parenchyma. The most frequently injured cranial nerve is the sixth cranial nerve (unilateral [16, 30] or bilateral [6, 14, 15, 19, 31–35]). Other affected nerves include the optic, oculomotor, trigeminal, facial, glossopharyngeal, and hypoglossal nerves. Patients may also exhibit hemiparesis or quadriparesis. The rare extreme cases include brain stem contusion with cardiorespiratory compromise [17–20, 36] and progressive hydrocephalus [19].

These hematomas may be overlooked on axial CT due to beam hardening artifacts in the posterior fossa [16], requiring reformatted CT images or MRI to elucidate the diagnosis and assess for ligamentous damage. Common etiologies can typically be inferred based on clinical presentation (history of trauma or presence of pituitary adenoma). Work-up for concurrent blunt traumatic vascular injury may be warranted. With no obvious mechanism, work-up for vascular pathology or coagulopathy should ensue [28]. The presence of ligamentous instability and brain injury or spinal cord injury will determine the appropriate management [11]. The possibility of brainstem compression or instability mandates initial close observation, reasonably within an ICU setting [30]. Although rare, the extra-axial hematoma can cause mass effect on the brainstem and cranial nerves, necessitating surgical evacuation [19, 35, 37, 38]. Of the 33 traumatic cases of rcEDH, twelve patients exhibited a cranial nerve palsy, five patients required surgical stabilization of the craniocervical junction [19, 38, 39], one patient required an external ventricular drain for progressive hydrocephalus [20], and six patients died. Of the 17 traumatic cases of rcSDH, no patient required surgical stabilization; one patient died. Of the 12 cases of pituitary apoplexy, all but 1 patient exhibited cranial nerve palsies; overall, surgical resection of the hemorrhagic pituitary adenoma has led to good outcomes [1, 24].

Except for the rare cases that lead to death [17, 18, 20, 28, 29, 37, 39], the majority of patients exhibit good outcomes with minimal long-term neurological deficits with conservative management. Tubbs et al. [39] noted no relationship between hematoma size and presenting symptoms; moreover, initial GCS did not correlate with outcomes. Hematoma appears to resolve within 2–11 weeks [14, 36, 39]. On admission, our patient exhibited bilateral 6th nerve palsies, consistent with prior reports. At discharge, 19 days after the accident, he exhibited intact eye movements. Flexion and extension films demonstrated no cervical instability, and his cervical spine was cleared.

## Conclusion

Retroclival hematoma may present after trauma. Most cases exhibit a benign clinical course with conservative management, but significant and profound morbidity and mortality have been reported. Prompt diagnosis with close observation is prudent. Surgical management is dictated based on the presence of hydrocephalus, brainstem compression, and occipito-cervical instability.
